# Non-Essential Element-Based Nanoparticles in Rice: Unraveling the Impacts of Yttrium Oxide and Zirconium Oxide Nanoparticles on Root Accumulation and Antioxidant Responses

**DOI:** 10.3390/plants15111727

**Published:** 2026-06-03

**Authors:** Boxuan Xie, Yukui Rui

**Affiliations:** 1 State Key Laboratory of Nutrient Use and Management, Beijing Key Laboratory of Farmland Soil Pollution Prevention and Remediation, College of Resources and Environmental Sciences, China Agricultural University, Beijing 100193, China; 13522830833@163.com; 2 Professor Workstation in Shanghe County, China Agricultural University, Shanghe, Jinan 251600, China; 3 Professor Workstation in Wuqiang County, China Agricultural University, Wuqiang, Hengshui 053300, China

**Keywords:** yttrium oxide nanoparticles, zirconium oxide nanoparticles, rice seedlings, growth traits, SPAD value, antioxidant enzymes, root accumulation

## Abstract

Nanotechnology has attracted increasing attention in agricultural and environmental research, but the biological effects and potential risks of nanoparticles based on non-essential elements remain insufficiently understood. This study investigated the physiological and biochemical responses of rice (*Oryza sativa* L.) seedlings to yttrium oxide nanoparticles (Y_2_O_3_ NPs) and zirconium oxide nanoparticles (ZrO_2_ NPs) at 5, 25, and 100 mg/L under hydroponic conditions. The results showed that neither Y_2_O_3_ nor ZrO_2_ NPs significantly affected visible growth traits or SPAD-based leaf chlorophyll status, suggesting that seedling morphology and leaf greenness remained relatively stable during exposure. However, both nanoparticles induced distinct biochemical responses. Y_2_O_3_ NPs caused root-level stress-like responses, including increased malondialdehyde (MDA) accumulation and suppressed peroxidase (POD) and catalase (CAT) activities under specific exposure conditions. In contrast, ZrO_2_ NPs were more closely associated with the activation of antioxidant defenses, particularly through enhanced POD activity and increased root CAT activity. Inductively coupled plasma mass spectrometry (ICP-MS) analysis further showed that Y and Zr were mainly retained in roots, with root Y reaching 5014.12–11,255.05 mg kg^−1^ dry weight (DW) under Y_2_O_3_ NP exposure and root Zr reaching 189.68 mg kg^−1^ DW under high-concentration ZrO_2_ NP exposure. Bio-transmission electron microscopy (bio-TEM) supported the root-dominant localization of nanoparticle-associated electron-dense aggregates. These findings indicate that Y_2_O_3_ and ZrO_2_ NPs exert material-specific effects on rice seedlings, with root accumulation and antioxidant regulation serving as more sensitive indicators than visible growth traits. However, further research is needed to clarify the long-term environmental fate of Y_2_O_3_ and ZrO_2_ NPs and to assess their potential ecological and food safety risks in agricultural systems.

## 1. Introduction

Rice (*Oryza sativa* L.), a major cereal crop and staple food for much of the global population, plays a vital role in global food security and agricultural sustainability [1,2]. Increasing demands for crop productivity and environmental safety have promoted the application of nanotechnology in agricultural research. Engineered nanoparticles, owing to their nanoscale size, large specific surface area, and unique physicochemical properties, can influence plant growth, nutrient uptake, and physiological processes. However, their effects are highly dependent on nanoparticle composition, dosage, exposure route, plant species, and the rhizosphere environment, resulting in either growth-promoting effects or phytotoxic responses under different conditions [3,4,5,6,7,8,9,10,11,12].

Yttrium oxide (Y_2_O_3_) and zirconium oxide (ZrO_2_) nanoparticles are metal oxide nanomaterials widely used in industrial, catalytic, optical, and biomedical fields. Their broad utilization raises concerns regarding potential environmental release throughout their life cycle, including production, application, and disposal. Unlike nanoparticles containing essential nutrient elements, Y and Zr are not essential for higher plants. Therefore, their biological effects, accumulation behavior, and potential risks in agricultural systems remain insufficiently understood. Previous studies have shown that plants are capable of taking up Y and Zr, and these elements tend to accumulate mainly in roots, raising concerns about their environmental fate and biological impacts in crop systems [13,14,15].

Plant responses to Y- and Zr-containing nanomaterials are strongly dependent on plant species, exposure concentration, and experimental conditions. Y_2_O_3_ nanoparticles were observed in cabbage tissues after root uptake and were mainly retained in belowground organs [16]. In rice seedlings grown under hydroponic conditions, Y_2_O_3_ nanoparticles and released Y^3+^ ions delayed germination at relatively high concentrations and affected root and shoot growth, indicating that rice can respond sensitively to Y-based nanoparticle exposure [17]. Similarly, Y_2_O_3_ nanoparticles were reported to inhibit growth and induce physiological and oxidative damage in tomato seedlings [18]. For Zr-based nanomaterials, ZrO_2_ nanoparticles caused toxicity and oxidative stress responses in Lemna minor [19], whereas in another experimental scenario, ZrO_2_ nanoparticles enhanced growth, photosynthetic pigments, and defense enzyme activities in tomato [20]. In rice hydroponic systems, engineered nanoparticles have also been used to evaluate nanoparticle-related effects on element uptake and physiological responses [21]. In addition, previous studies from our group have shown that rice can respond sensitively to nanoparticle exposure, with changes in uptake, growth, stress regulation, and nutrient-related physiological processes under different exposure scenarios [22,23,24,25,26,27,28].

Comparative studies have further demonstrated that plant responses to nanomaterials depend strongly on plant type and physiological characteristics, with C3 crops such as rice often showing distinct responses compared with C4 plants [29]. Moreover, phytotoxic effects of metal oxide nanoparticles have been reported in other crop systems, indicating that nanoparticle-induced responses vary with material type, plant species, and exposure conditions [30]. These findings suggest that visible growth responses alone may not fully reflect the internal physiological and biochemical disturbances induced by engineered nanoparticles, especially for non-essential element-based nanoparticles.

Despite these advances, comparative information on Y_2_O_3_ and ZrO_2_ nanoparticles in rice remains very limited, particularly regarding whether these two non-essential element-based nanoparticles mainly affect visible growth performance or induce more subtle physiological and biochemical responses. Integrated evaluations combining growth traits, SPAD-based leaf chlorophyll status, malondialdehyde (MDA) content, peroxidase (POD) activity, catalase (CAT) activity, Y/Zr accumulation, and tissue localization are still scarce in rice seedlings. Therefore, in this study, rice seedlings were exposed to different concentrations of Y_2_O_3_ and ZrO_2_ nanoparticles, and their effects on seedling growth, SPAD-based leaf chlorophyll status, oxidative stress responses, antioxidant enzyme activities, Y/Zr accumulation, and ultrastructural localization revealed by bio-transmission electron microscopy (bio-TEM) were systematically investigated. This study aimed to compare the response patterns of rice seedlings to two representative non-essential element-based nanoparticles and to provide experimental evidence for evaluating their potential agricultural and environmental risks.

## 2. Materials and Methods

Yttrium oxide nanoparticles (Y_2_O_3_ NPs) and zirconium oxide nanoparticles (ZrO_2_ NPs) used in this study were purchased from Zhengzhou Convergence Chemical Company (Zhengzhou, China). Both nanomaterials had a purity greater than 99.9%, were supplied as dry powders, and were used without further chemical modification. All other chemicals were of analytical grade. The morphology and primary particle characteristics of the nanoparticles were examined using transmission electron microscopy (TEM). For TEM analysis, the nanoparticle powders were ultrasonically dispersed in deionized water, dropped onto copper grids, and dried before observation. The hydrodynamic diameter, polydispersity index (PdI), and zeta potential of the nanoparticle suspensions were measured using a Zetasizer Nano ZS 90 particle size analyzer (Malvern Panalytical Ltd., Malvern, UK), with deionized water as the dispersing medium.

### 2.1. Experimental Design

Seeds of rice (*Oryza sativa* L. cv. Nipponbare) with uniform size were surface-sterilized with 0.5% NaClO for 30 min and then rinsed thoroughly with deionized water. The sterilized seeds were germinated at 26 °C under a 12 h light/12 h dark photoperiod for 9 days. Subsequently, uniformly developed seedlings were selected for the hydroponic experiment.

A hydroponic system was used in this study to provide a controlled exposure environment, ensure direct contact between rice roots and nanoparticle suspensions, and reduce the confounding effects caused by nanoparticle adsorption, transformation, and heterogeneity in soil. Therefore, this system was suitable for evaluating the early-stage physiological and biochemical responses of rice seedlings to Y_2_O_3_ and ZrO_2_ nanoparticles under controlled conditions.

The experiment was arranged as a completely randomized design with seven treatments: a blank control without nanoparticles (CK), three Y_2_O_3_ NP treatments at 5, 25, and 100 mg/L, and three ZrO_2_ NP treatments at 5, 25, and 100 mg/L. The selected concentrations represented low, intermediate, and high exposure levels, respectively. The low concentration of 5 mg/L was selected because previous studies have shown that Y_2_O_3_ NPs can affect plant responses at low concentrations, including 1–10 mg/L in rice seedlings and 1–5 mg/L in tomato seedlings [17,18]. The intermediate concentration of 25 mg/L was used to bridge the low- and high-exposure levels. The high concentration of 100 mg/L was included as a high-exposure scenario to evaluate potential toxicological risks, as 100 mg/L-level nanoparticle treatments have been used in previous Y_2_O_3_, ZrO_2_, and rice hydroponic nanoparticle studies [17,18,20,21].

For each treatment, the required amounts of Y_2_O_3_ or ZrO_2_ nanoparticle powder were weighed and dispersed in Hoagland nutrient solution. The suspensions were ultrasonicated for 15 min before use and mixed thoroughly before being added to the culture bottles.

Each bottle contained three rice seedlings and was considered one biological replicate. Four biological replicates were established for each treatment, resulting in 12 seedlings per treatment for growth observation and measurement. During the 30-day hydroponic cultivation period, the nutrient solution was not completely replaced. Instead, fresh Hoagland nutrient solution was added daily to restore the initial volume. Before the seedlings were returned to the bottles, the exposure medium was ultrasonicated and thoroughly mixed to maintain nanoparticle dispersion.

### 2.2. Measurement of Growth Traits

At harvest, shoot height and root length were measured with a ruler. Shoot height was measured from the base of the shoot to the tip of the longest leaf, whereas root length was measured from the root base to the tip of the longest root. After length measurements, shoots and roots were separated with scissors and weighed using an analytical balance to determine shoot fresh weight and root fresh weight.

### 2.3. Measurement of SPAD Value

SPAD values were measured immediately after harvest using a SPAD meter (IN-YL03, Shandong Laiyin Optoelectronic Technology Co., Ltd., Weifang, China). For each biological replicate, one representative healthy leaf was selected from the seedlings grown in the same bottle, and the measurement was performed at the middle portion of the leaf blade. The same leaf position was used across all treatments to reduce measurement variation. Each bottle was considered one biological replicate.

### 2.4. Determination of MDA and Antioxidant Enzymes

Fresh shoot and root tissues were collected separately from each biological replicate. For each replicate, 0.15 g of fresh tissue was weighed and homogenized with 600 μL of phosphate-buffered saline (PBS) in a pre-cooled mortar. The homogenates were centrifuged at 8000 rpm for 10 min at 4 °C, and the supernatants were collected as crude enzyme extracts. The malondialdehyde (MDA) content and the enzymatic activities of peroxidase (POD) and catalase (CAT) were then analyzed using commercial assay kits (Nanjing Jiancheng Bioengineering Institute, Nanjing, China).

The MDA content was determined using the thiobarbituric acid (TBA) reaction. In this assay, MDA reacts with TBA to form a colored product with a maximum absorbance at 532 nm, and the MDA content was calculated according to the kit protocol.

The POD activity was measured based on the POD-catalyzed reaction with hydrogen peroxide. Enzyme activity was determined by recording the absorbance at 420 nm according to the manufacturer’s instructions.

The CAT activity was determined based on the decomposition of H_2_O_2_. Residual H_2_O_2_ reacts with ammonium molybdate to form a yellow complex, and the absorbance at 405 nm was used to calculate CAT activity.

Absorbance measurements were performed using a microplate reader, and all results were calculated and normalized according to the manufacturer’s protocols.

### 2.5. Determination of Y and Zr Contents

Yttrium (Y) and zirconium (Zr) contents in rice tissues were analyzed by inductively coupled plasma mass spectrometry (ICP-MS, Thermo Scientific, Waltham, MA, USA). After harvest, shoot and root samples were collected separately and dried to constant weight.

The dried plant samples were subjected to acid digestion before ICP-MS determination. The samples were transferred into digestion tubes, followed by the addition of 3–4 mL of high-purity nitric acid and approximately 1 mL of hydrogen peroxide. The digestion tubes were then heated on an electric heating plate at 180 °C for approximately 30 min to decompose the plant matrix. After digestion, the acid was evaporated until the residual solution volume was reduced to approximately 1 mL. The remaining solution was diluted to a fixed volume with ultrapure water and thoroughly mixed prior to ICP-MS analysis. Sample digestion and ICP-MS measurement were performed by Zhongke Baice Company (Beijing, China).

### 2.6. Bio-TEM Observation of Rice Tissues

Bio-transmission electron microscopy (bio-TEM) was used to examine the ultrastructural localization of nanoparticle-associated electron-dense aggregates in rice tissues. Based on the ICP-MS accumulation results, tissues from the CK, 100 mg/L Y_2_O_3_ NP, and 100 mg/L ZrO_2_ NP treatments were selected for bio-TEM observation.

The samples were prepared following a standard ultrathin-section transmission electron microscopy procedure. Fixed samples were rinsed three times with 0.1 M phosphate buffer and then post-fixed with 1% osmium tetroxide. After post-fixation, the samples were rinsed again with phosphate buffer and dehydrated through a graded ethanol series. The samples were subsequently treated with absolute ethanol and transitioned with acetone. Resin infiltration was then performed using acetone–embedding resin mixtures, followed by pure resin infiltration overnight. The infiltrated samples were embedded and polymerized at 70 °C overnight.

Ultrathin sections of approximately 70–90 nm were obtained using an ultramicrotome and stained with lead citrate and uranyl acetate. The stained sections were then observed using a low-voltage transmission electron microscope. The bio-TEM sample preparation and observation were performed by Zhongke Baice Company, Beijing, China.

### 2.7. Data Analysis

Data are presented as mean ± standard deviation (SD) from four biological replicates (n = 4). Statistical analysis was performed using IBM SPSS Statistics 27.0. Differences among treatments were analyzed by one-way analysis of variance (ANOVA), followed by Tukey’s multiple comparisons test. In the figures, only statistically significant differences between nanoparticle-treated groups and the control group (CK) are indicated with asterisks: * *p* < 0.05, ** *p* < 0.01, *** *p* < 0.001, and **** *p* < 0.0001.

For ICP-MS analysis, heatmap visualization was performed using Z-score-standardized ICP-MS data.

## 3. Results and Discussion

### 3.1. Characterization of Y_2_O_3_ and ZrO_2_ Nanoparticles

**Figure 1:**
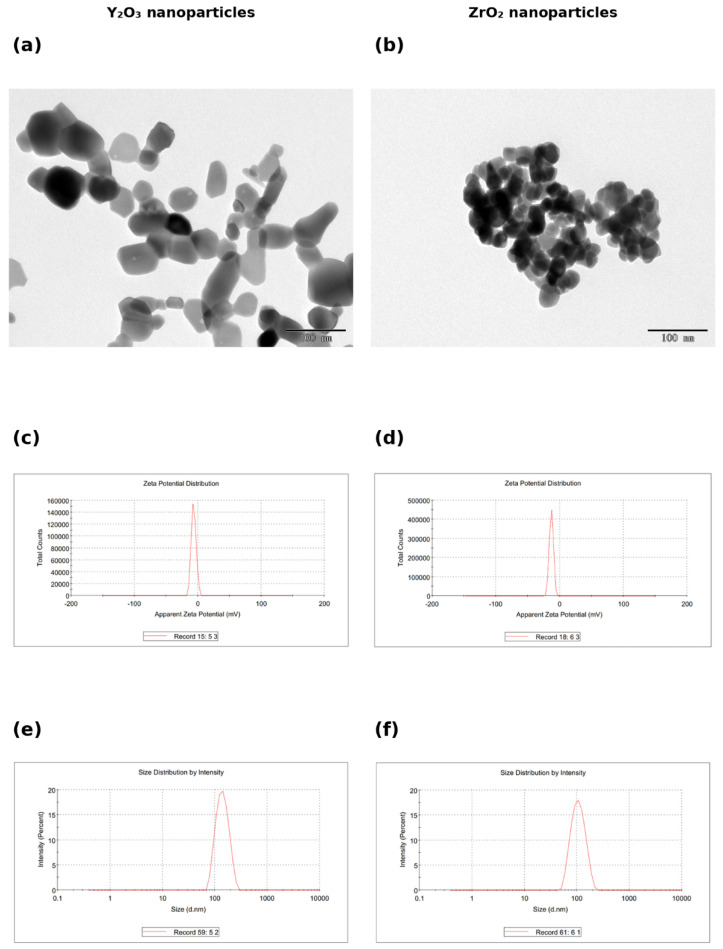
Characterization of Y_2_O_3_ and ZrO_2_ nanoparticles. TEM electron micrographs of Y_2_O_3_ NPs (**a**) and ZrO_2_ NPs (**b**). Hydrodynamic diameter distributions of Y_2_O_3_ NPs (**c**) and ZrO_2_ NPs (**d**). Zeta potential distributions of Y_2_O_3_ NPs (**e**) and ZrO_2_ NPs (**f**).

The physicochemical properties of Y_2_O_3_ and ZrO_2_ nanoparticles were characterized before hydroponic exposure, as these properties are closely related to their behavior in exposure media and their subsequent interactions with plant roots. As shown in Figure 1 and Table 1, both nanoparticles exhibited typical nanoscale morphology under transmission electron microscopy (TEM). After ultrasonic dispersion, the hydrodynamic diameter of Y_2_O_3_ NPs was 138.87 ± 1.93 nm, with a polydispersity index (PdI) of 0.208 ± 0.016. In comparison, ZrO_2_ NPs showed a smaller hydrodynamic diameter of 100.29 ± 0.97 nm and a lower PdI of 0.096 ± 0.020, indicating a relatively narrower particle size distribution in aqueous medium.

The zeta potentials of Y_2_O_3_ and ZrO_2_ NPs were −6.33 ± 0.31 mV and −12.77 ± 0.23 mV, respectively. The more negative zeta potential of ZrO_2_ NPs suggested relatively stronger electrostatic repulsion among particles compared with Y_2_O_3_ NPs. Taken together, ZrO_2_ NPs showed a smaller hydrodynamic diameter, lower PdI, and more negative zeta potential than Y_2_O_3_ NPs under the tested dispersion conditions, suggesting relatively better dispersion behavior. These differences may influence nanoparticle behavior in the hydroponic exposure medium, including dispersion stability, aggregation, sedimentation, and contact with rice roots. In contrast, the larger hydrodynamic diameter, higher PdI, and less negative zeta potential of Y_2_O_3_ NPs may indicate a stronger tendency toward aggregation and sedimentation, potentially favoring their retention near the root surface. However, because the exposure medium contained Hoagland nutrient solution rather than pure water, nutrient ions may further modify nanoparticle aggregation, surface charge, and root interactions during hydroponic exposure. Therefore, the physicochemical characterization of Y_2_O_3_ and ZrO_2_ NPs provides an important basis for interpreting their biological effects and for evaluating the environmental safety of non-essential element-based nanoparticles in agricultural systems.

### 3.2. Effects of Y_2_O_3_ and ZrO_2_ Nanoparticles on Growth Traits of Rice Seedlings

**Figure 2:**
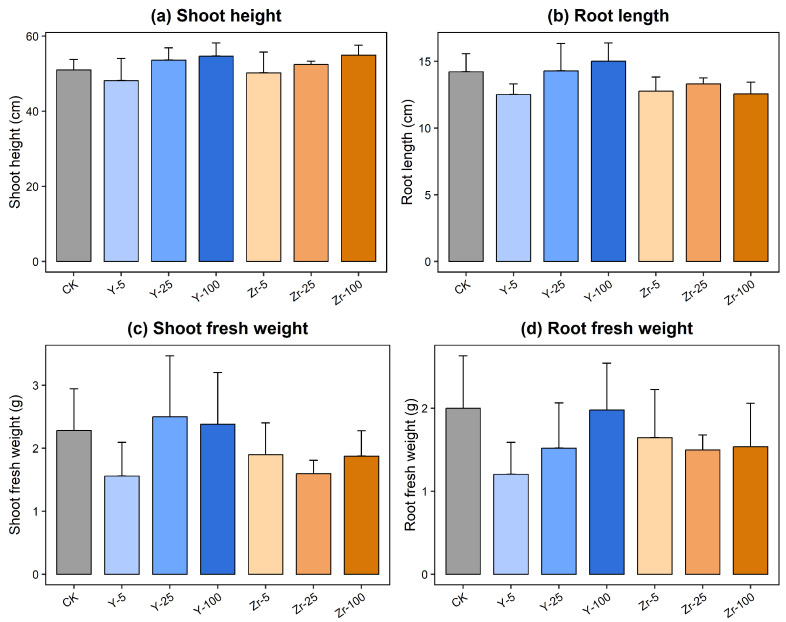
Effects of Y_2_O_3_ and ZrO_2_ nanoparticles on growth traits of rice seedlings. Data are presented as mean ± SD (n = 4 biological replicates). No statistically significant differences were detected between nanoparticle-treated groups and the control group (CK) according to one-way ANOVA followed by Tukey’s multiple comparisons test.

To evaluate the visible growth responses of rice seedlings to Y_2_O_3_ and ZrO_2_ nanoparticle exposure, shoot height, root length, shoot fresh weight, and root fresh weight were measured at harvest (Figure 2). Overall, no statistically significant changes in these growth traits were detected between nanoparticle-treated seedlings and the control group under the tested hydroponic conditions. Although minor numerical variations were observed among treatments, these changes did not show a consistent concentration-dependent inhibitory or stimulatory pattern.

Shoot height remained relatively stable across all treatments (Figure 2a). Compared with the CK, Y_2_O_3_ NP treatments showed slight numerical changes, with relatively higher values at 25 and 100 mg/L. ZrO_2_ NP treatments also showed only minor fluctuations, and the shoot height under 100 mg/L ZrO_2_ NPs was numerically higher than that of the CK. However, these differences were not statistically significant, indicating that shoot elongation was not markedly affected by either Y_2_O_3_ or ZrO_2_ NP exposure.

Root length also showed limited variation among treatments (Figure 2b). The root length of seedlings exposed to 25 and 100 mg/L Y_2_O_3_ NPs was numerically similar to or slightly higher than that of the CK, whereas ZrO_2_ NP treatments showed slight decreases relative to the CK. However, these changes were not significant and did not indicate a clear dose-dependent response.

For biomass-related traits, shoot fresh weight and root fresh weight showed larger numerical fluctuations than shoot height and root length, but no significant differences were detected among treatments (Figure 2c,d). Shoot fresh weight was numerically higher under 25 and 100 mg/L Y_2_O_3_ NPs than under CK, whereas the ZrO_2_ NP treatments showed relatively small decreases or fluctuations (Figure 2c). Root fresh weight showed a similar pattern of treatment-dependent variation, with relatively higher values under 100 mg/L Y_2_O_3_ NPs but lower values under several other nanoparticle treatments compared with CK (Figure 2d). These variations were not statistically significant, suggesting that biomass accumulation remained relatively stable under the present exposure conditions.

**Figure 3:**
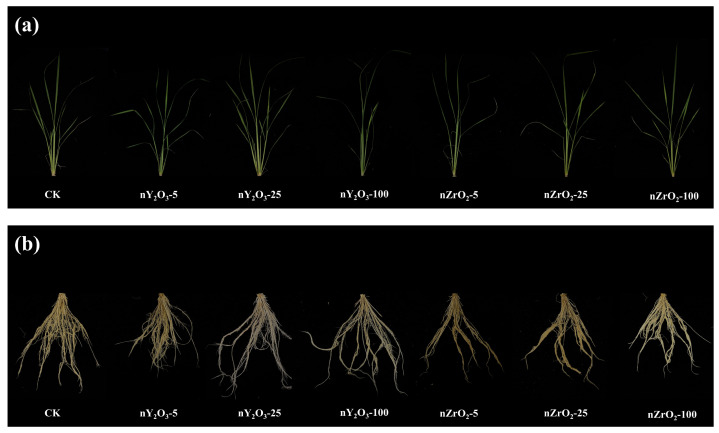
Representative photographs of rice seedlings under different Y_2_O_3_ and ZrO_2_ nanoparticle treatments at harvest. (**a**) Shoots and (**b**) roots.

Representative photographs of shoots and roots were used to provide a visual overview of seedling morphology at harvest (Figure 3). The shoot photographs showed broadly comparable aboveground morphology among CK and nanoparticle-treated seedlings (Figure 3a). The root photographs showed visible variation in root architecture among treatments, but these images were not used for quantitative comparison (Figure 3b). Therefore, the interpretation of growth responses was based mainly on the measured growth traits shown in Figure 2. Taken together, these results indicate that Y_2_O_3_ and ZrO_2_ NPs did not induce significant changes in visible growth traits of rice seedlings under the tested conditions.

### 3.3. Effects of Y_2_O_3_ and ZrO_2_ Nanoparticles on Leaf Chlorophyll Status as Indicated by SPAD Values

**Figure 4:**
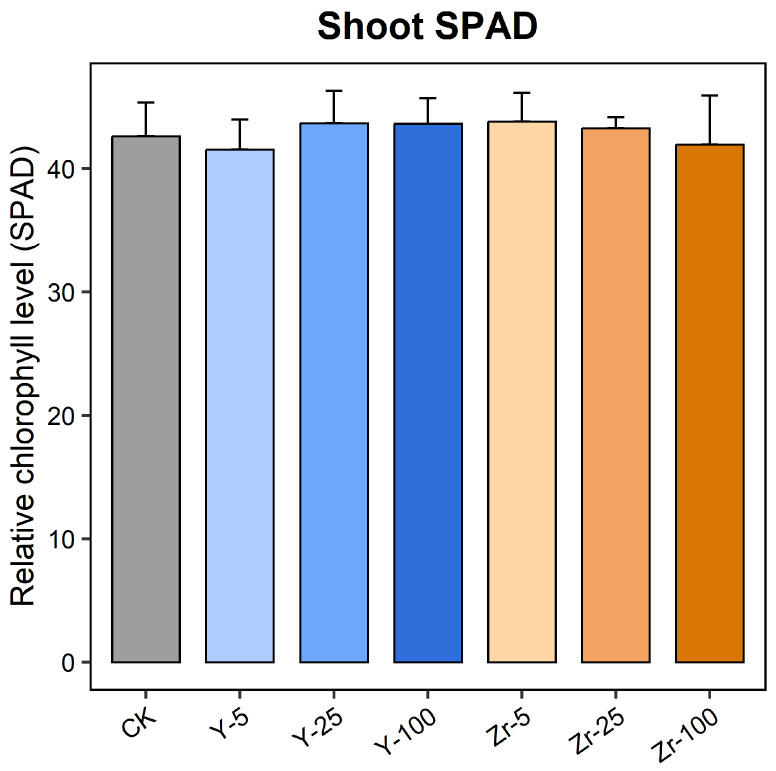
Effects of Y_2_O_3_ and ZrO_2_ nanoparticles on leaf chlorophyll status as indicated by SPAD values. Data are presented as mean ± SD (n = 4 biological replicates). No statistically significant differences were detected between nanoparticle-treated groups and the control group (CK) according to one-way ANOVA followed by Tukey’s multiple comparisons test.

SPAD value is a nondestructive relative index that reflects leaf optical properties and is commonly used to indirectly evaluate leaf chlorophyll status, although it does not represent chlorophyll content determined by pigment extraction [31]. Under the present experimental conditions, neither Y_2_O_3_ nor ZrO_2_ NPs significantly altered SPAD values in rice leaves, although slight numerical variations were observed among treatments (Figure 4).

Compared with the CK, Y_2_O_3_ NP treatments caused only minor changes in SPAD values, with a slight numerical decrease at 5 mg/L and small increases at 25 and 100 mg/L. Similarly, ZrO_2_ NP treatments slightly increased SPAD values at 5 and 25 mg/L but caused a slight decrease at 100 mg/L. These changes remained within a narrow range, from −2.5% to 2.8% relative to the CK, indicating that the leaf chlorophyll status of rice seedlings remained generally stable under Y_2_O_3_ and ZrO_2_ NP exposure. Taken together, these results suggest that Y_2_O_3_ and ZrO_2_ NPs did not significantly affect the SPAD-based chlorophyll status of rice leaves under the tested hydroponic conditions. This finding was consistent with the relatively weak treatment effects observed for most visible growth traits, indicating that leaf greenness and morphological traits were not highly sensitive to Y_2_O_3_ and ZrO_2_ NP exposure in this study. However, previous studies have shown that chlorophyll-related responses to nanomaterials can vary among plant species and physiological types, including differences between C3 and C4 plants [29]. Therefore, stable SPAD values do not necessarily indicate the absence of nanoparticle-induced physiological stress. Oxidative damage and antioxidant enzyme activities were further examined to determine whether these nanoparticles induced more sensitive biochemical responses in rice seedlings.

### 3.4. Effects of Y_2_O_3_ and ZrO_2_ Nanoparticles on Oxidative Stress and Antioxidant Enzyme Activities in Rice Seedlings

Reactive oxygen species (ROS) overproduction can disturb cellular redox homeostasis and induce oxidative stress in plants, thereby affecting plant growth and development [32,33]. Malondialdehyde (MDA), a product of membrane lipid peroxidation, is commonly used as an indicator of oxidative damage in plant tissues [33,34]. Therefore, MDA content was measured to evaluate lipid peroxidation in rice seedlings exposed to Y_2_O_3_ and ZrO_2_ nanoparticles.

**Figure 5:**
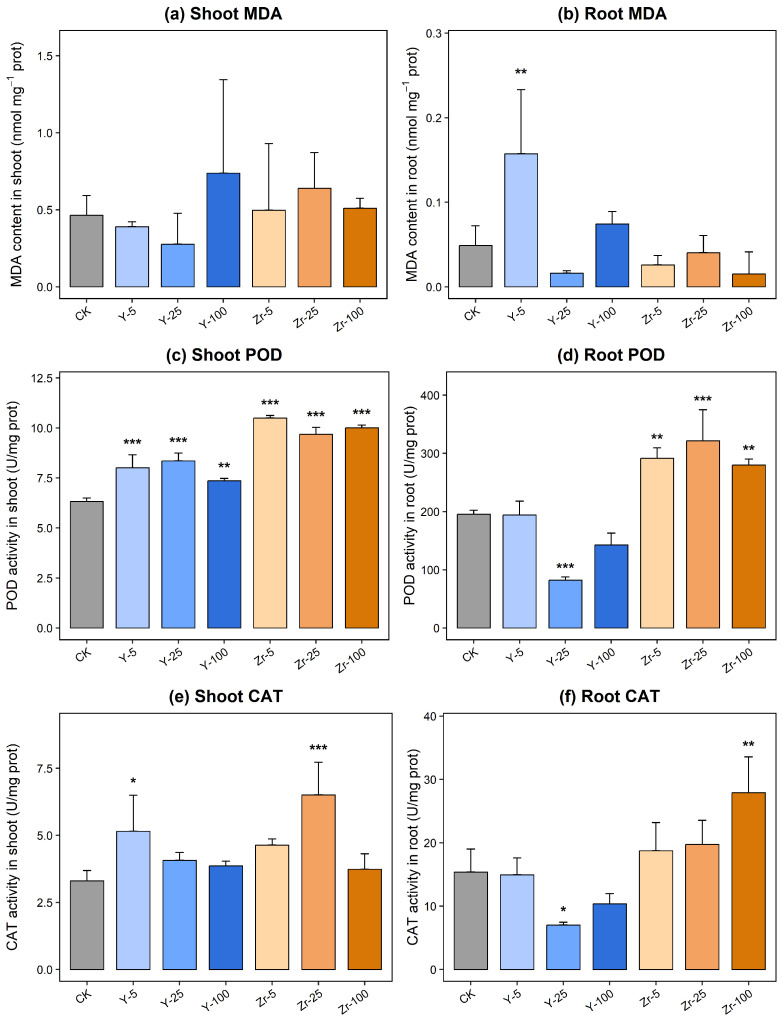
Effects of Y_2_O_3_ and ZrO_2_ nanoparticles on oxidative stress and antioxidant enzyme activities in rice seedlings. (**a**,**b**) MDA content in shoots and roots, respectively; (**c**,**d**) POD activity in shoots and roots, respectively; and (**e**,**f**) CAT activity in shoots and roots, respectively. Data are presented as mean ± SD (n = 4 biological replicates). Asterisks indicate statistically significant differences between nanoparticle-treated groups and the control group (CK): * *p* < 0.05, ** *p* < 0.01, and *** *p* < 0.001.

As shown in Figure 5a,b, the effects of nanoparticle exposure on MDA content were tissue-dependent. In shoots, MDA content showed numerical variation among treatments, but no statistically significant differences were detected between nanoparticle-treated groups and the CK (Figure 5a). This result suggested that lipid peroxidation in aboveground tissues remained relatively stable under the tested exposure conditions. In roots, however, MDA content responded more clearly to nanoparticle exposure (Figure 5b). Compared with the CK, 5 mg/L Y_2_O_3_ NPs significantly increased root MDA content, indicating enhanced lipid peroxidation at this concentration. In contrast, ZrO_2_ NP treatments showed lower numerical MDA values in roots than the CK, although these decreases were not statistically significant. These results indicate that nanoparticle-induced lipid peroxidation responses were more evident in roots than in shoots, which may be associated with the direct exposure of roots to nanoparticle suspensions.

Plants rely on antioxidant defense systems to scavenge excessive ROS and maintain cellular redox balance [33,35]. Peroxidase (POD) and catalase (CAT) are important antioxidant enzymes involved in peroxide detoxification and stress alleviation [35,36]. Therefore, POD and CAT activities were further measured to evaluate the antioxidant responses of rice seedlings to Y_2_O_3_ and ZrO_2_ NP exposure.

POD activity showed clear material- and tissue-dependent response patterns (Figure 5c,d). In shoots, all Y_2_O_3_ and ZrO_2_ NP treatments significantly increased POD activity compared with the CK, indicating that shoot POD activity was responsive to both nanoparticles (Figure 5c). The increase was particularly evident under ZrO_2_ NP treatments, which showed higher POD activities than most Y_2_O_3_ NP treatments. In roots, the response patterns of the two nanoparticles differed substantially (Figure 5d). Y_2_O_3_ NPs caused no significant change at 5 mg/L, but significantly reduced root POD activity at 25 mg/L. By contrast, ZrO_2_ NPs significantly increased root POD activity at all tested concentrations, with the strongest response observed at 25 mg/L. These results suggest that ZrO_2_ NP exposure was associated with enhanced POD-mediated antioxidant responses, whereas Y_2_O_3_ NPs showed an inhibitory effect on root POD activity at a specific concentration. Similar nanoparticle-induced regulation of antioxidant metabolism has also been reported in crop plants, indicating that antioxidant enzymes are sensitive biochemical indicators of nanoparticle exposure [6,7,26,28,29].

CAT activity exhibited a more selective response than POD activity (Figure 5e,f). In shoots, 5 mg/L Y_2_O_3_ NPs and 25 mg/L ZrO_2_ NPs significantly increased CAT activity compared with the CK, whereas the other treatments caused only nonsignificant numerical changes (Figure 5e). In roots, Y_2_O_3_ and ZrO_2_ NPs again showed different response patterns (Figure 5f). The 25 mg/L Y_2_O_3_ NP treatment significantly decreased root CAT activity, while 100 mg/L ZrO_2_ NPs significantly increased root CAT activity. Although ZrO_2_ NPs at 5 and 25 mg/L also showed numerically higher root CAT activity than CK, these differences were not statistically significant. These results indicate that CAT activity was regulated in a concentration- and tissue-dependent manner, with ZrO_2_ NPs tending to enhance antioxidant enzyme activity, particularly at higher concentration in roots, whereas Y_2_O_3_ NPs suppressed root CAT activity at 25 mg/L.

Taken together, Y_2_O_3_ and ZrO_2_ NPs induced more evident biochemical responses than visible growth traits or SPAD-based chlorophyll status in rice seedlings. Root-level biochemical responses were more pronounced than shoot responses, especially for MDA content, POD activity, and CAT activity. This pattern was consistent with the direct exposure of roots to nanoparticle suspensions under hydroponic conditions and suggested that roots were the primary sites of nanoparticle-induced biochemical regulation. Moreover, the two nanoparticles showed distinct regulatory patterns: ZrO_2_ NP exposure was generally associated with enhanced POD activity in both shoots and roots and increased root CAT activity at the highest concentration, whereas Y_2_O_3_ NP exposure induced root lipid peroxidation at 5 mg/L and suppressed root POD and CAT activities at 25 mg/L. Since H_2_O_2_ and O_2_^−^ were not directly quantified in this study, MDA content and antioxidant enzyme activities were used as indirect indicators of oxidative status.

### 3.5. Y and Zr Accumulation in Rice Seedlings

**Figure 6:**
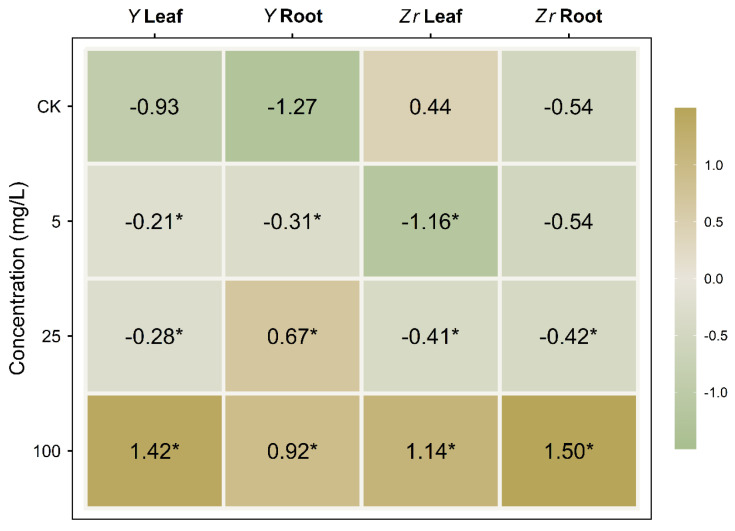
Heatmap of Y and Zr accumulation in rice tissues. Y Leaf/Root represent Y_2_O_3_ NP treatments, and Zr Leaf/Root represent ZrO_2_ NP treatments. Values are column-wise Z-score-standardized ICP-MS data. * *p* < 0.05 vs. CK.

To further evaluate whether the root-level biochemical responses were associated with the accumulation and distribution of the two non-essential elements, Y and Zr contents in rice shoots and roots were determined by inductively coupled plasma mass spectrometry (ICP-MS). The original ICP-MS data were expressed as mg kg^−1^ dry weight (DW) and used for quantitative interpretation. For a more intuitive comparison of relative accumulation patterns among treatments and tissues, the measured Y and Zr concentrations were further standardized using Z-score normalization and visualized as a heatmap (Figure 6). The Z-score-standardized values reflected the relative enrichment or depletion of each element across treatments, whereas the original concentrations were retained to describe the actual accumulation levels.

As shown in Figure 6, Y accumulation displayed a clear root-dominant pattern under Y_2_O_3_ NP exposure. In shoots, Y content showed only limited changes after Y_2_O_3_ NP treatment. In contrast, markedly higher Y levels were detected in roots, particularly under the 25 and 100 mg/L Y_2_O_3_ NP treatments. The measured ICP-MS data showed that root Y content reached 5014.12–11,255.05 mg kg^−1^ DW under Y_2_O_3_ NP exposure, corresponding to approximately 50.6–113.5 times that of the CK. These results indicate that Y derived from Y_2_O_3_ NP exposure was predominantly retained in root tissues, with limited upward translocation to shoots.

For Zr, a similar root-dominant accumulation pattern was observed under ZrO_2_ NP exposure, although the overall accumulation level was lower than that of Y. Shoot Zr content remained at a relatively low level across ZrO_2_ NP treatments, whereas root Zr content increased with increasing exposure concentration. Under the 100 mg/L ZrO_2_ NP treatment, root Zr content reached 189.68 mg kg^−1^ DW, approximately 58.5 times that of the CK. In shoots, only limited Zr accumulation was detected, suggesting restricted upward transport of Zr-associated materials under the tested hydroponic conditions.

Trace Y and Zr signals were also detected in the CK and non-corresponding nanoparticle treatments. These low background signals may be related to naturally occurring trace elemental levels in rice tissues, nutrient solution, or the experimental system, as well as the high sensitivity of ICP-MS. Nevertheless, treatment-induced accumulation was most evident in the corresponding nanoparticle exposure groups, particularly root Y under Y_2_O_3_ NP treatments and root Zr under high-concentration ZrO_2_ NP treatment.

Taken together, Y_2_O_3_ and ZrO_2_ NP treatments resulted in distinct accumulation patterns of the corresponding non-essential elements in rice seedlings. Y derived from Y_2_O_3_ NP exposure accumulated predominantly in roots, with only limited changes observed in shoots, indicating strong root retention and restricted upward translocation. Zr accumulation also showed a root-dominant pattern under ZrO_2_ NP exposure, although the overall accumulation level was much lower than that of Y. These results suggest that different non-essential element-based nanoparticles may exhibit different accumulation intensities and tissue distribution behaviors in rice seedlings. The preferential retention of Y and Zr in roots may be closely related to direct root exposure to nanoparticle suspensions in the hydroponic system and may partly explain the more evident root-level biochemical responses observed in this study. Considering that Y and Zr are not essential nutrients for plant growth, their accumulation in crop tissues should be carefully evaluated in future studies, particularly with regard to long-term exposure, food safety, and environmental risk assessment. Previous studies have also shown that the distribution, transformation, and root-associated interactions of engineered nanomaterials in plant systems are important factors determining their biological effects and environmental implications [12,37].

### 3.6. Bio-TEM Evidence Supporting the Differential Physiological Responses of Rice Seedlings to Y_2_O_3_ and ZrO_2_ Nanoparticles

**Figure 7:**
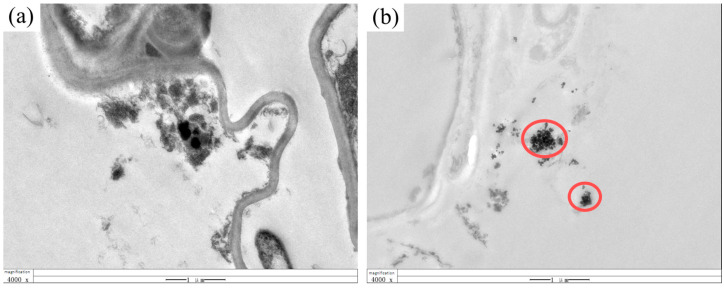
Bio-TEM images of rice roots under CK and 100 mg/L Y_2_O_3_ NP treatment. (**a**) CK root; (**b**) Y_2_O_3 _NP-treated root. Red circles indicate electron-dense aggregates. Scale bars: 1 μm.

**Figure 8:**
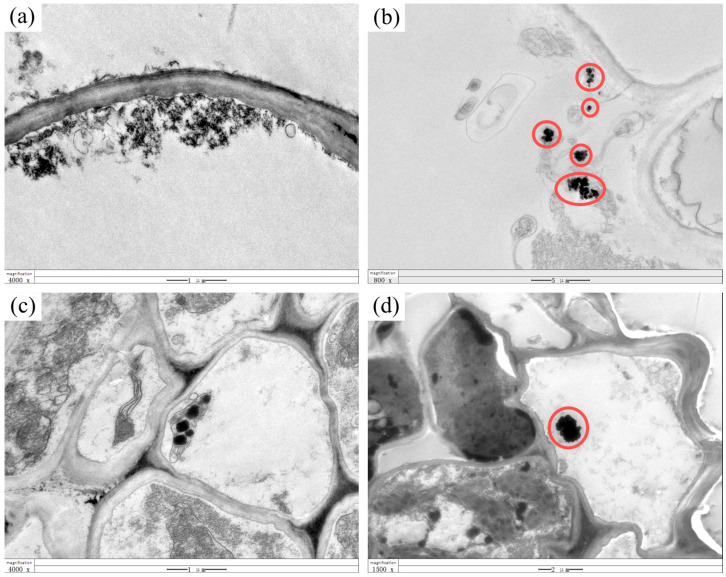
Bio-TEM images of rice root and leaf sections under CK and 100 mg/L ZrO_2_NP treatment. (**a**) CK root; (**b**) ZrO_2_ NP-treated root; (**c**) CK leaf; and (**d**) ZrO_2_ NP-treated leaf. Red circles indicate electron-dense aggregates. Scale bars: 1 μm in (**a**), 5 μm in (**b**), 1 μm in (**c**), and 2 μm in (**d**).

Based on the ICP-MS results showing root-dominant Y and Zr accumulation, bio-transmission electron microscopy (bio-TEM) was further used to examine the ultrastructural localization of nanoparticle-associated electron-dense aggregates in rice tissues. As shown in Figure 7 and Figure 8, no obvious electron-dense particulate aggregates were observed in the corresponding tissues of the CK group. In contrast, electron-dense aggregates were observed in root tissues after exposure to both Y_2_O_3_ and ZrO_2_ NPs, providing ultrastructural evidence consistent with the root-dominant accumulation pattern revealed by ICP-MS.

The tissue localization patterns differed between the two nanoparticles. Under Y_2_O_3_ NP exposure, electron-dense aggregates were mainly observed in root tissues, whereas no obvious nanoparticle-associated aggregates were detected in the examined leaf sections (Figure 7). This observation suggests that Y-associated materials were largely retained in roots under the present hydroponic conditions. By comparison, ZrO_2_ NP exposure also resulted in electron-dense aggregates mainly in roots, while limited electron-dense aggregates were observed in leaves at the highest exposure concentration (Figure 8). This pattern suggests that Zr-associated materials may have undergone limited upward transport from roots to shoots. Similar root retention, transformation, and internal localization of engineered nanomaterials in plants have also been reported in previous studies from our group [37,38,39].

These tissue localization patterns were consistent with the elemental accumulation results and may help explain the different biochemical responses induced by Y_2_O_3_ and ZrO_2_ NPs. The preferential retention of Y-associated materials in roots was consistent with the stronger root-level oxidative response observed under specific Y_2_O_3_ NP treatments, including increased root MDA content and suppressed POD and CAT activities. In contrast, the relatively smaller hydrodynamic diameter, lower PdI, and more negative zeta potential of ZrO_2_ NPs suggested better dispersion behavior, which may have contributed to their more homogeneous interaction with rice roots and limited upward transport. This was consistent with the biochemical results showing enhanced POD activity and increased root CAT activity under ZrO_2_ NP exposure. Since root-associated interfaces are key sites regulating nanoparticle retention, transformation, and plant responses, the distinct localization patterns observed here may be an important factor contributing to the material-specific physiological responses of rice seedlings [12].

Taken together, the bio-TEM observations supported the root-dominant retention of nanoparticle-associated materials and provided ultrastructural evidence linking tissue localization patterns with the differential physiological and biochemical responses of rice seedlings to Y_2_O_3_ and ZrO_2_ NP exposure.

## 4. Conclusions

This study evaluated the physiological and biochemical responses of rice seedlings to Y_2_O_3_ and ZrO_2_ nanoparticles under hydroponic conditions. The results showed that both nanoparticles had limited effects on visible growth traits and SPAD-based leaf chlorophyll status, indicating that seedling morphology and leaf chlorophyll status remained relatively stable during the exposure period. However, oxidative stress indicators, antioxidant enzyme activities, elemental accumulation, and tissue localization showed clearer treatment responses. Y_2_O_3_ NPs were more closely associated with root-level stress-like responses, including enhanced lipid peroxidation and suppression of antioxidant enzyme activities under specific exposure conditions, whereas ZrO_2_ NPs tended to activate antioxidant defenses, as reflected by increased POD and CAT activities and relatively lower root lipid peroxidation. These results indicate that Y_2_O_3_ and ZrO_2_ NPs induced material-specific physiological responses, and that internal biochemical indicators and root accumulation were more sensitive than visible growth traits for evaluating the effects of non-essential element-based nanoparticles on rice seedlings.

From an environmental and agricultural safety perspective, the accumulation of Y and Zr in crop tissues deserves further attention because these elements are not essential nutrients for plant growth. The root-dominant accumulation pattern observed in this study suggests that roots may act as the main retention site for Y- and Zr-associated materials under hydroponic exposure, but their long-term fate, transformation, persistence, and bioavailability in soil–plant systems remain unclear. In real agricultural environments, nanoparticle behavior may be further influenced by soil minerals, organic matter, root exudates, redox conditions, and rhizosphere microorganisms, which could alter their mobility, transformation, plant uptake, and ecological effects [12,40]. Therefore, future studies should extend beyond short-term hydroponic exposure and evaluate the long-term accumulation, trophic transfer potential, food safety implications, and ecological risks of Y_2_O_3_ and ZrO_2_ NPs under soil culture and field conditions. In addition, combining long-term exposure experiments, field-scale assessments, advanced elemental imaging, and molecular biological approaches would help clarify their uptake pathways, transformation processes, and biological effects in crop systems. Such information is essential for establishing a more comprehensive risk assessment framework and for guiding the responsible management of non-essential element-based nanoparticles in agricultural systems [11].

## Figures and Tables

**Table 1:** Hydrodynamic size, polydispersity index (PdI), and zeta potential of Y_2_O_3_ and ZrO_2_ nanoparticles in deionized water.

Material	Z-Average (nm)	PdI	Zeta Potential (mV)
Y_2_O_3_	138.87 ± 1.93	0.208 ± 0.016	−6.33 ± 0.31
ZrO_2_	100.29 ± 0.97	0.096 ± 0.020	−12.77 ± 0.23

Data are presented as mean ± SD (n = 3).

## Data Availability

The original contributions presented in this study are included in the article. Further inquiries can be directed to the corresponding author.
